# Patient Symptoms and Stress Testing After Elective Percutaneous Coronary Intervention in the Veterans Affairs Health Care System

**DOI:** 10.1001/jamanetworkopen.2022.17704

**Published:** 2022-06-21

**Authors:** Vinay Kini, Monica Parks, Wenhui Liu, Stephen W. Waldo, P. Michael Ho, Steven M. Bradley, Paul L. Hess

**Affiliations:** 1 Division of Cardiology, Weill Cornell Medical College, New York, New York; 2 Division of Cardiology, University of Colorado Anschutz Medical Campus, Aurora; 3 Veterans Affairs Eastern Colorado Healthcare System, Aurora; 4 Veterans Affairs Clinical Assessment Reporting and Tracking Program, Veterans Health Administration Office of Quality and Patient Safety, Washington, DC; 5 Minneapolis Heart Institute, Minneapolis, Minnesota

## Abstract

**Importance:**

Up to 60% of patients in the US receive a stress test within 2 years of percutaneous coronary intervention (PCI), prompting concerns about the possible overuse of stress testing.

**Objective:**

To examine the proportion of patients who underwent stress testing within 2 years of elective PCI, proportion of patients who had symptoms that were consistent with coronary artery disease (CAD), timing of stress testing, and site-level variation in stress testing among symptomatic and asymptomatic patients.

**Design, Setting, and Participants:**

This cohort study used administrative claims data and clinical records from the US Department of Veterans Affairs (VA) Clinical Assessment, Reporting, and Tracking program. Patients who underwent stress testing within 2 years of elective PCI for stable CAD between November 1, 2013, and October 31, 2015, at 64 VA facilities were included in the analysis. Patients who received stress testing for staging purposes, cardiac rehabilitation evaluation, or preoperative testing before high-risk surgery were excluded. Data were analyzed from June to December 2020.

**Main Outcomes and Measures:**

The main outcome was the proportion of patients who underwent stress testing and had symptoms that were consistent with obstructive CAD, using definitions from the 2013 clinical practice guideline (Multimodality Appropriate Use Criteria for the Detection and Risk Assessment of Stable Ischemic Heart Disease). Secondary outcomes were the timing of stress testing (assessed using a cumulative incidence curve) and site-level variation in stress testing (assessed using multilevel logistic regression models).

**Results:**

A total of 3705 consecutive patients (mean [SD] age 66.3 [7.6] years; 3656 men [98.7%]; 437 Black individuals [11.8%], 3175 White individuals [85.7%], and 93 individuals [2.5%] of other races and ethnicities [Asian, Hispanic or Latinx, or unknown]) had elective PCI. Of these patients, 916 (24.7%) received a stress test within 2 years, among whom 730 (79.7%) had symptoms that were consistent with obstructive CAD at the time of stress testing. Visual inspection of a cumulative incidence curve for stress testing showed no rapid increases in stress testing at 6 months or 1 year after PCI, which might coincide with routine clinical visits. The proportion of symptomatic patients who underwent stress testing at each VA site ranged from 67.7% to 100%, with no significant site-level variation in stress testing.

**Conclusions and Relevance:**

Results of this study suggest that most veterans who underwent stress testing within 2 years after elective PCI had symptoms that were consistent with obstructive CAD. Therefore, measuring low-value stress testing using only administrative claims data may overestimate its prevalence, and concerns about overuse of post-PCI stress testing may be overstated.

## Key Points

**Question:**

What proportion of patients who underwent stress testing within 2 years of elective percutaneous coronary intervention (PCI) showed symptoms that were consistent with obstructive coronary artery disease?

**Findings:**

In this cohort study of 3705 patients who underwent elective PCI, 24.7% of patients underwent stress testing within 2 years, and 79.7% of those who had stress testing had symptoms that were suggestive of obstructive coronary artery disease.

**Meaning:**

Findings of this study suggest that measuring low-value stress testing using administrative claims data alone may overestimate its prevalence and that overuse of stress testing 2 years after PCI may be an overstated concern.

## Teaser

This cohort study assesses the incidence and timing of stress testing among patients with or without symptoms of obstructive coronary artery disease.

## Introduction

Stress testing among patients within 2 years of percutaneous coronary intervention (PCI) is common, ranging from 40% to 60% in national cohort studies.^1,2,3,4^ The 2013 clinical guideline (Multimodality Appropriate Use Criteria for the Detection and Risk Assessment of Stable Ischemic Heart Disease) states that stress testing within 2 years of PCI is indicated (ie, appropriate) for patients who have symptoms consistent with obstructive coronary artery disease (CAD).^5^ However, previous studies have shown that (1) only 20% of patients report recurrent symptoms after PCI, suggesting that the rate of stress testing may exceed the rate that is explainable by symptoms^6^; (2) there is wide variation in rates of stress testing between hospitals and regions that is unexplainable by patient characteristics or differences in outcomes^7^; and (3) there are rapid increases in stress testing rates at 6 months and 1 year after coronary revascularization, suggesting that routine testing may be occurring at follow-up clinical visits.^1^ These findings have raised concerns that a substantial proportion of stress tests performed within 2 years after PCI may be considered low value (ie, not indicated by the 2013 guideline).^8^

Several previous studies have examined the national prevalence and factors associated with use of low-value stress testing among patients with CAD using administrative claims data (eg, Medicare fee-for-service, US Department of Veterans Affairs [VA], and commercial insurance databases).^9,10,11^ However, because claims data do not contain information on patient symptoms, the proportion of stress tests that may have been clinically indicated (ie, prompted by symptoms consistent with obstructive CAD) is not well understood. If a large proportion of patients have symptoms at the time of stress testing, it may suggest that claims-based measures of low-value stress testing overestimate its prevalence and that concerns about overuse are exaggerated. Conversely, if only a small proportion of patients have symptoms or if there are sites that perform stress testing in asymptomatic patients at substantially higher rates than in other patients, concerns about overuse may be justified and claims-based measures should continue to be used to assess rates of low-value post-PCI stress testing. Accordingly, in this study, we used data from a national cohort of veterans to examine (1) the proportion of patients who underwent stress testing within 2 years of elective PCI, (2) the proportion of patients who had symptoms that were consistent with CAD, (3) the timing of stress testing, and (4) the site-level variation in stress testing among symptomatic and asymptomatic patients.

## Methods

This retrospective cohort study was approved by the Colorado Multiple Institution Review Board, which waived the informed consent requirement given the minimal risk exposure. We followed the Strengthening the Reporting of Observational Studies in Epidemiology (STROBE) reporting guideline.

### Data Sources and Study Population

We used data from the VA Clinical Assessment, Reporting, and Tracking program, which is responsible for monitoring the quality and safety of invasive cardiac procedures throughout the VA health care system. This program uses a software application embedded in the VA electronic health record to collect patient and procedural data at the point of care for all PCIs performed at VA hospitals. These data are linked to the VA electronic health record, allowing for linkage to longitudinal data on health care service utilization, medication use, and outcomes.^12^

We identified all patients who underwent elective PCI for stable CAD between November 1, 2013, and October 31, 2015, at 64 VA facilities. Patients who received catheterization for acute coronary syndrome, preoperative assessment, valvular heart disease, or cardiomyopathy were excluded. Percutaneous coronary intervention data were linked to cardiac catheterization report documentation and clinical pharmacy data. Patient race and ethnicity was obtained from the VA Corporate Data Warehouse, which contains the most recent race and ethnicity information entered into the Veterans Health Information Systems and Technology Architecture system, and included Black, White, and other [Asian, Hispanic or Latinx, or unknown] race and ethnicity categories. In the event that multiple entries were found, priority was given to the race and ethnicity that were self-reported or reported by a proxy.

### Identification of Stress Tests and Symptoms

Trained medical record abstractors identified all modalities of stress testing (stress electrocardiography [ECG], stress echocardiography, nuclear single-photon emission computed tomography [SPECT], and coronary computed tomographic angiography [CCTA]) performed from 60 days to 2 years after PCI. To ensure that all stress tests, including those administered outside of the VA health care system, were captured, we reviewed notes from cardiology and primary care clinic visits as well as stress testing reports that were entered manually into the VA electronic health record. Stress tests conducted within 60 days after PCI were excluded because they may have been performed for staging (and therefore clinically indicated even in the absence of symptoms).

Using medical record abstraction, we excluded stress tests performed in patients with an exercise prescription, to establish baseline exercise capacity before cardiac rehabilitation was initiated, or in patients who were undergoing preoperative evaluation before high-risk (any vascular or solid organ transplant) surgery. Stress test results were obtained from clinical reports and were identified as positive if any ischemia was detected on ECG, echocardiography, or nuclear SPECT or if more than 50% left-main coronary stenosis or more than 70% stenosis in any other vessel was detected on CCTA.

We used definitions from the 2013 guideline (Multimodality Appropriate Use Criteria for the Detection and Risk Assessment of Stable Ischemic Heart Disease) to identify symptoms that may have prompted stress testing.^5^ Trained personnel abstracted symptoms from individual patient records (ie, medical record review). Symptoms were defined as any constellation of clinical findings (including chest pain, tightness, or burning; dyspnea; or reduced or worsening effort tolerance) that the clinician deemed consistent with obstructive CAD. Given the nature of real-world documentation, standardized reports of anginal symptoms using instruments, such as the Canadian Cardiovascular Society classification of angina pectoris severity or the Seattle Angina Questionnaire, were not available in most records. Symptoms were therefore dichotomized as either present (symptomatic) or absent (asymptomatic). To assess interrater reliability, 330 records were randomly selected for abstraction, which yielded a Cohen κ of 0.95 (95% CI, 0.92-0.97).

### Statistical Analysis

We evaluated the overall proportion of patients who underwent stress testing after PCI as well as the proportion of symptomatic and asymptomatic patients among this group. We compared the demographic and clinical characteristics of these patients as well as the types and results of the stress tests using χ^2^ tests for categorical data and unpaired, 2-tailed *t* tests for continuous data. We examined the timing of stress testing (between 60 days and 2 years after PCI) using visual inspection of a cumulative incidence curve.

We also examined facility-level variation in stress testing. By clinical site, we summarized with medians and IQRs the proportion of stress tests administered to symptomatic patients. We ascertained site-level variations in the estimated proportion of stress tests performed among symptomatic patients using multilevel logistic regression models with a random site-level intercept. We used a Markov chain Monte Carlo method with 500 burn-in iterations and 10 000 simulations to obtain estimated rates of stress testing and 95% CIs.

Analyses were conducted using SAS statistical software, version 9.4 (SAS Institute). *P* values were 2-sided, and statistical significance was set at *P* = .05. Data were analyzed from June to December 2020.

## Results

A total of 3705 patients (mean [SD] age 66.3 [7.6] years; 3656 men [98.7%] and 49 women [1.3%]; 437 Black individuals [11.8%], 3175 White individuals [85.7%], and 93 individuals [2.5%] of other races and ethnicities [Asian, Hispanic or Latinx, or unknown]) who underwent elective PCI for stable CAD were included in this study. Among these patients, 916 (24.7%) underwent stress testing between 60 days and 2 years after PCI. Of the 916 patients, 730 (79.7%) had symptoms that were suggestive of CAD and 65 (7.1%) had no symptoms; for 121 patients (13.2%), we were unable to ascertain the symptom status from medical record review. Most of the symptoms reported were chest pain (74.3%; 591 patients) and dyspnea (50.1%; 398 patients).

We compared the demographic and clinical characteristics of those patients whose symptom status could be confirmed (n = 795) (Table 1). A greater proportion of symptomatic patients than asymptomatic patients received more antianginal medication prescriptions (≥2 antianginal drugs, 30.2% vs 10.7%; *P* = .03) and were more likely to have single-vessel obstructive CAD (60.1% vs 40.0%; *P* = .002) on their index CCTA.

**Table 1.** Characteristics of Patients Undergoing Stress Testing After Percutaneous Coronary Intervention by Symptom Status

Characteristic	No. (%)			*P* value
	Overall	Asymptomatic patients	Symptomatic patients	
No. of patients	795 (100)	65 (8.2)	730 (91.8)	NA
Age, mean (SD), y	65.7 (7.7)	65.1 (7.2)	65.7 (7.7)	.56
Sex				
Female	23 (2.9)	1 (0)	22 (3.0)	.46
Male	772 (97.1)	64 (98.5)	707 (96.8)	
Race and ethnicity^a^				
Black	90 (11.3)	14 (21.5)	76 (10.4)	.09
White	679 (85.4)	51 (78.4)	628 (86.0)	
Other^b^	26 (3.3)	0	26 (3.3)	
No. of antianginal medications prescribed				
0	166 (20.8)	17 (26.2)	149 (20.4)	.03
1	397 (49.9)	41 (63.1)	356 (48.8)	
2	201 (25.2)	6 (9.2)	195 (26.7)	
3	24 (3.0)	1 (1.5)	23 (3.2)	
4	1 (0)	0	1 (0)	
Coronary anatomy, obstructive				
1-Vessel	461 (58.0)	26 (40.0)	435 (59.6)	.002
2-Vessel	168 (21.1)	24 (36.9)	144 (19.7)	
3-Vessel or left-main	128 (16.1)	14 (21.5)	114 (15.6)	
Not available	32 (4.0)	1 (1.5)	31 (4.2)	
Smoking history	559 (70.3)	46 (70.8)	513 (70.3)	>.99
Hypertension	759 (95.5)	63 (96.9)	696 (95.3)	.78
Hyperlipidemia	767 (96.5)	61 (93.8)	706 (96.7)	.40
Diabetes	425 (53.5)	39 (60.0)	386 (52.8)	.33
Family history of CAD	184 (23.1)	19 (29.2)	165 (22.6)	.29
Previous MI	334 (42.0)	26 (40.0)	308 (42.2)	.83
Heart failure	200 (25.2)	25 (38.5)	175 (24.0)	.02
Previous stroke or TIA	70 (8.8)	5 (7.7)	65 (8.9)	.92
Dialysis	32 (4.0)	6 (9.2)	26 (3.6)	.06
CKD	171 (21.5)	19 (29.2)	152 (20.8)	.16
PAD	183 (23.0)	17 (26.2)	166 (22.7)	.64

Abbreviations: CAD, coronary artery disease; CKD, chronic kidney disease; MI, myocardial infarction; NA, not applicable; PAD, peripheral arterial disease; TIA, transient ischemic attack.

^a^ Race and ethnicity were obtained from the Veterans Affairs Corporate Data Warehouse and were self-reported or reported by a proxy.

^b^ Other included Asian, Hispanic or Latinx, and unknown race and ethnicity.

We also compared stress test characteristics for symptomatic and asymptomatic patients (Table 2). A greater proportion of patients with post-PCI symptoms compared with those without such symptoms underwent nuclear SPECT (87.1% vs 76.9%; *P* = .02), whereas a smaller proportion underwent exercise ECG without accompanying imaging (6.8% vs 18.5%; *P* = .02). The proportion of stress tests with ischemia-positive results was similar among symptomatic and asymptomatic patients (45.1% vs 36.9%; *P* = .39).

**Table 2.** Patient Symptoms, Stress Test Modalities, and Stress Test Results

Variable	No. (%)			*P* value
	Overall	Asymptomatic patients	Symptomatic patients	
No. of patients	795 (100)	65 (8.2)	730 (91.8)	NA
Symptoms				
Chest pain	591 (74.3)	0	591 (81.0)	<.001
Dyspnea	398 (50.1)	0	398 (54.5)	<.001
Type of stress test				
Echocardiography	44 (5.5)	3 (4.6)	41 (5.6)	.02
Nuclear SPECT	686 (86.3)	50 (76.9)	636 (87.1)	
Exercise ECG	62 (8.0)	12 (18.5)	50 (6.8)	
CCTA	1 (0)	0	1 (0)	
Post-PCI stress test result				
Negative	394 (49.6)	38 (58.5)	356 (48.8)	.39
Positive	353 (44.4)	24 (36.9)	329 (45.1)	
Nondiagnostic	25 (3.1)	2 (3.1)	23 (3.2)	
Unavailable	18 (2.3)	1 (1.5)	17 (2.3)	

Abbreviations: CCTA, coronary computed tomographic angiography; ECG, electrocardiography; NA, not applicable; PCI, percutaneous coronary intervention; SPECT, single-photon emission computed tomography.

**Figure 1.**
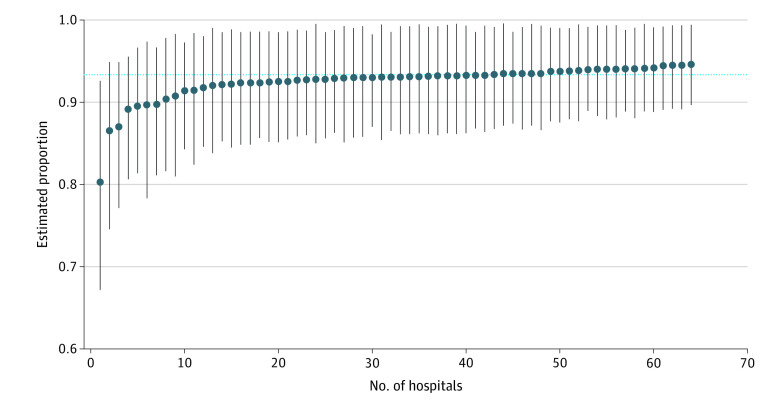
Site-Level Variation in the Estimated Proportion of Stress Testing Among Symptomatic Patients Minimal site-level variation in the proportion of symptomatic patients who received stress tests within 2 years of percutaneous coronary intervention was observed using multilevel regression models.

We also examined variation in post-PCI stress testing across the 64 clinical sites. The number of post-PCI stress tests conducted at each site ranged from 2 to 32, with a median (IQR) of 17 (11-27). The proportion of symptomatic patients who underwent stress testing at each site ranged from 67.7% to 100%, with a median (IQR) of 93.3% (89.9%-100%). At 33 sites, all of the post-PCI stress tests performed were in symptomatic patients. There was minimal site-level variation in the estimated proportion of stress tests administered to symptomatic patients (Figure 1).

**Figure 2.**
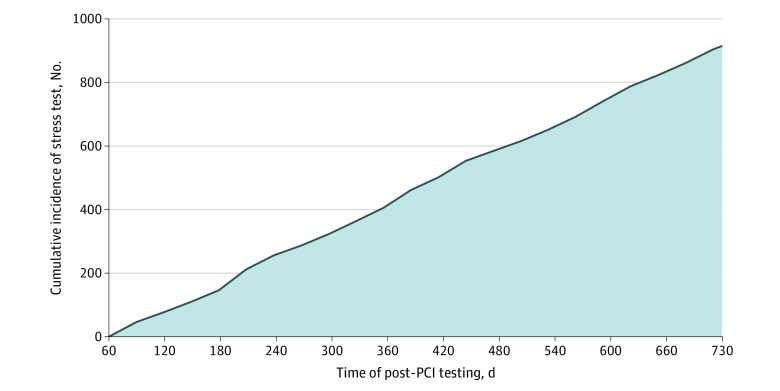
Cumulative Incidence of Stress Testing After Elective Percutaneous Coronary Intervention (PCI) A steady linear increase in the incidence of stress testing was observed, without rapid increases in stress testing at usual follow-up clinical visits (ie, 6 months and 1 year).

In exploring the timing of post-PCI stress testing, we found an overall steady increase in stress testing between 60 days and 2 years after PCI (Figure 2). No rapid increases in stress testing were observed at any time point.

## Discussion

In a large, national cohort of patients who had elective PCI at VA hospitals, we found that 24.7% of patients underwent stress testing between 60 days and 2 years after PCI and that 79.7% of those patients reported symptoms that were consistent with obstructive CAD at the time of testing. There was minimal site-level variation in the proportion of stress tests that were prompted by symptoms, and there were no increases in stress testing rates at 6 months or 1 year after PCI that would coincide with the usual timing of follow-up clinical visits. Patients with symptoms were more likely to receive an imaging stress test, and the proportion of stress tests with ischemia-positive results were similar between symptomatic and asymptomatic patients.

### Concerns About Overuse

According to the 2013 clinical practice guideline, stress testing within 2 years of PCI is rarely appropriate in patients without symptoms that are suggestive of obstructive CAD (ie, an ischemic equivalent).^5^ Previous studies have shown that 40% to 60% of patients undergo post-PCI stress testing,^1,2,3,4^ raising concerns about the possible overuse of stress tests. However, the present study found that most patients had symptoms that were consistent with obstructive CAD, which may have prompted stress testing in accordance with the 2013 clinical practice guideline.^5^ The patient population also had a lower rate of overall stress testing (24.7%), likely because previous studies included patients who received PCI for acute coronary syndrome, whereas the present study included only patients who received elective PCI. In addition, we found minimal site-level variation in stress testing among symptomatic patients, in contrast to previous studies that reported substantial site-level variation in overall use of post-PCI stress tests.^1,2,4,13^ This discrepancy may be attributable to differences in patient population (symptomatic patients after elective PCI in the present study), sample size (smaller in the present study), or payment model (integrated settings often have less variation than fee-for-service settings because of less fragmented care).^13^ Moreover, we found no rapid increases in stress testing at 6 months or 1 year after PCI, indicating that stress tests were not ordered routinely during follow-up clinical visits but rather may have been prompted by symptoms as the 2013 guideline suggests.^5^ Thus, the findings of this study suggest that concerns about overuse of stress testing in the 2 years after elective PCI may be overstated, at least in the VA health care system but possibly in other health care settings as well.

### Use of Clinical Data in Adjudicating Stress Testing Value

Previous studies used administrative claims data to estimate the proportion of stress testing that is considered to be low value (ie, provides no net benefit) among patients with stable ischemic heart disease. One study suggested that the rate of low-value stress testing may range between 1 and 8 per 1000 Medicare fee-for-service beneficiaries annually and cost between $212 million and $2.8 billion.^9^ Other studies similarly used administrative claims data to examine whether there are differences in use of low-value stress testing between health care systems, health care payers, and payment models (fee-for-service vs value-based).^10,11,14,15,16^ In contrast, the present study supplemented administrative claims data with detailed medical record review to understand whether stress testing may have been performed in accordance with the clinical guideline. In this context, the results suggest that without clinical data to ascertain patient symptoms, it is likely that a large proportion of stress tests that were considered to be low value in previous studies may have been clinically indicated; therefore, the true prevalence of low-value stress testing may be lower than previously reported. Future studies are needed that use clinical data to adjudicate the value of stress tests and procedures that may be indicated because of symptoms or other data, which are not generally available in administrative claims data.

### Stress Test Modalities and Results

The 2013 clinical practice guideline recommends using a stress imaging test, rather than exercise ECG alone, in patients with symptoms after PCI.^5^ Previous studies of post-PCI stress testing identified substantial nationwide variation in utilization of stress test modalities, although none have described these modalities in relation to symptom status.^17^ In this study, we found that although stress imaging tests (nuclear SPECT and stress echocardiography) were the dominant modalities overall, symptomatic patients were more likely than asymptomatic patients to receive a stress imaging test. This finding may be attributable to a higher pretest probability of obstructive CAD in symptomatic patients or with symptomatic patients having limited exercise tolerance, leading clinicians to order more imaging-based stress tests. Moreover, we found that the proportion of stress tests with an ischemia-positive result was substantial (about 40%) but was not significantly different between symptomatic and asymptomatic patients. Previous studies found a lower yield of abnormal stress test results, demonstrating a markedly low yield of ischemia-positive stress test results after PCI.^18,19^ A potential explanation for this finding is the differences in patient populations between the present and past studies, with a substantial proportion of veterans having obstructive CAD. Future studies evaluating the use of subsequent repeat CCTA or PCI would be helpful in elucidating the reasons for this finding.

### Limitations

This study has several limitations. First, the assessment of symptoms was cross-sectional, and thus we were unable to comment on whether there were changes in symptoms that prompted stress testing. Future studies should examine symptom burden over time and its implications for the use of post-PCI stress testing. Second, this study was retrospective and, as such, was subjected to possible residual confounding as well as loss to follow-up and clinical documentation biases; that is, we were unable to ascertain symptoms for 13.2% of patients, and some patients may have undergone stress testing or may have had their symptoms documented outside of the VA health care system, beyond the records that were available to us. Third, although we randomly selected 330 records for independent review to maximize interrater reliability, medical documentation of reported angina may not fully represent the actual symptom burden after PCI.^20^ Fourth, many VA sites performed only a small number of post-PCI stress tests, which may have limited our ability to detect significant variation in stress testing. Fifth, we did not include data on subsequent CCTA or PCI after stress testing. Sixth, the results may not be fully generalizable because the study cohort was composed predominantly of White male patients; future studies should be conducted in non-VA settings. Seventh, there could have been differences in the availability of stress test modalities across sites. Although such differences were unlikely to have altered the proportion of patients who underwent post-PCI stress testing, they could have affected the proportion of patients undergoing a specific type of stress test or the proportion of stress tests with abnormal results.

## Conclusions

Among the patients who had elective PCI at VA hospitals across the US, approximately 25% underwent stress testing within 2 years of PCI. Approximately 80% of those who had stress testing reported symptoms that were consistent with obstructive CAD. The results of this cohort study suggest that concerns about the overuse of stress testing in the 2 years after PCI may be overstated, although further studies in non-VA settings are needed to examine the generalizability of the findings. The results highlight the importance of supplementing administrative claims data with clinical data to accurately ascertain the prevalence of low-value stress testing.
